# 

**Published:** 1842-12

**Authors:** 


### WESTERN JOURNAL

OF

### MEDICINE AND SURGERY.

EDITORS.

---

DANIEL DRAKE, M. D.,

PROFESSOR OF PATHOLOGICAL ANATOMY AND CLINICAL MEDICINE

IN THE MEDICAL INSTITUTE OF LOUISVILLE:

LUNSFORD P. YANDELL, M. D.,

PROFESSOR OF CHEMISTRY AND PHARMACY IN THE SAME:

THOMAS W. COLESCOTT, M. D.

### INDEX TO VOL. VI.

A
Abdominal tumors, 342.
Abuse of calomel by southern physicians, 474.
Alumni Society of the University of New York, transactions of the, 472.
Amaurosis, strychnia in, 397.
American Journal and Library of Dental Science, 61.
American Medicine before the Revolution, 149.
Amphoric rhonchus, researches into physical causes of, 145.
Amputations, circular and flap, comparative advantages of, 63.
Anatomy and Surgery, fall course of lectures on, 160.
Anatomy of the spleen, Bourgery on, 297.
Anatomy of fatty liver, 143.
Animal Chemistry, Liebig's, 342.
Antrum of Highmore, wound of, 186.
Asylum Journal, 470.
Auscultation, improvements in medicine from, 1, 80, 81.

B
Baron Larrey, 384.
Barry's Researches in Embryology, 257.
Bartlett on typhoid and typhus fever, 458.
Bell's Medical Library, 238.
Blood-globules, their formation and use, 463.
Bourgery on the anatomy of the spleen, 297.
Brain, compression of, 321.

C.
Cancerous affections of the womb, 380.
Carroll on scarlatina anginosa, 327.
Cartilages of the ribs, removal of—cure, 192.
Case of enormously enlarged stomach, 293.
Cases, miscellaneous, by Dr. Hogg, 252.
Catalogue and Circular of the Medical Department of Laporte University, 460.
Cataract, subconjunctival operation for, 473.
Calomel, abuse of by southern physicians, 474.
Chailley and Devilliers on auscultation in pregnancy, 392.
Chemistry, Liebig's Animal, 342.
" Kane's Elements of, 398.
Children at the breast, dentition of, 215.
Chronic spinitis, case of, 323.
Circular and flap amputations, comparative advantages of, 63.
Clinical lecture on delirium tremens, 196.
College of professional teachers, 79.
College, medical, report on, 235, 313.
Coloration of the bones by madder, 465.
Comparative frequency of tuberculous disease, 67.
Compression of the brain, 321.
Contraction of the fingers, 223.
Correction, 80.
Counter-irritants, 305.
Creasote, styptic effects of, 383.
Croton oil, external application of, 468.

D
Death of Dr. Samuel Hogg, 151.
Delirium tremens, clinical lecture on, 196.
Dentition of children at the breast, 215.
Devilliers and Chailley on auscultation in pregnancy, 392.
Diabetes mellitus, 69.
Diagnosis of gonorrhœa in accusations of rape, 147.
Diaphragm, rupture of in a horse, 470.
Diplomas, Latin, 236.
Diseases of Stark County, Ohio, 156.
Diseases of Rutherford County, Tennessee, 426.
Disease of the kidney, 462.
Disease, tuberculous, comparative frequency of, 67.

Discoloration of the skin from nitrate of silver, removal of, 395.
Division of muscles in scrivener's spasm, 190.
Donne on wound of antrum of highmore, 186.
Double uterus, physiological observations on, 467.
Dunglison's Medical Library, 238.

E

Effects of the times on the profession, 189.
Elements of Chemistry, Kane's, 398.
Eements of Surgery, Gross's edition of Liston's, 453.
Embryology, Barry's researches in, 257.
Enlarged stomach, 293.
Examination, post mortem, of an old soldier, 393.
Experiments on mesmerism, 71.
" kiesteine, 206.
Expulsion of a mass of hair from the uterus, 217.
External application of croton oil, 468.
Extract of belladonna, in sciatica, 394.
Eyelid, cysts of, 311.

F

Face, gun-shot wound of, 230.
Facts and conjectures on the trembles and milk-sickness, 181.
Fall course of lectures on anatomy and surgery, 160.
Fatty degeneration of liver, 144.
" " minute anatomy of, 143.
Femoral aneurism, ligature of external iliac for, 299.
Fever, yellow, of Natchez and the southwest, 161.
Fever, typhoid and typhus, Bartlett on, 458.
Fever, typhoid, Louis on, 386.
Fingers. permanent contraction of, 223.
Flap and circular amputations, compared, 63.
Fluor spar, 313.
Formation and use of blood-globules, 463.
Fractures, old, division of tendons in, 147.
Fracture of the tibia—necrosis—resection, 65.

G

Gonorrhœa, diagnosis of, in accusations of rape, 147.
Gross' edition of Liston's Elements of Surgery, 453.
Gun-shot wound of the face, 230.

H

Hair, mass of, expelled from the uterus, 217.
Hemorrhage, secondary, after amputation, 63.
History, diagnosis and treatment of typhoid and typhus fever, 458.
Hogg, Dr. Samuel, death of. 151.
" " miscellaneous cases of, 252.
Homœopathy, 319.
Hydrocele, new mode of treating, 194.
Hydrophobia, state of the blood in, 469.

I

Incipient stage of cancerous affections of the womb, 380.
Incomes of professional men in London, 296.
Influence of the nerves on muscular irritability, 193.
Injuries of the superior extremities, 241.
Injury to cartilages of the ribs—removal—cure, 192.
Improvements in practical medicine from auscultation, 1, 80, 81.
Instruction, private medical, 160, 239.
Introduction of syphilis into Scotland, 196.
Invalids of the south, summer resort for, 401.

J

Journal and Library of Dental Science, 61.
Journal of Medicine and Surgery, New England Quarterly, 59.

K

Kane's Elements of Chemistry, 398.
Kidney, disease of the, 462.
Kiesteine, experiments on, 206.
Kreasote, styptic effect of, 383.

L

Lakes of the north as a summer residence, 401.
Lancet, Western, 158.
Laporte University, catalogue and circular of, 460.
Larrey, death of, 384.
Latin diplomas, 236.
Lectures on anatomy and surgery, fall course of, 160.
Lecture on delirium tremens, 196.
Liebig's Animal Chemistry, 342.
Ligature of the primitive iliac, 390.
" external iliac, 299.
Lime moxa, 194.
Liston's Elements of Surgery, 453.
Liver, fatty degeneration of, 143, 144.
Louis on typhoid fever, 386.

M

Madder, coloration of the bones by, 465.
Maleolus internus, avulsion of, with fracture of tibia, 65.
Mass of hair, expelled from the uterus, 217.
Medicine, American, state of before the Revolution, 149.
Medicine and Surgery, New England Quarterly Journal of, 59.
Medical instruction, private, 160, 239.
Medical Society of Tennessee, 150.
Medical College, report on, 235, 313.
" appointments, 239.
Metallic tinkling, physical causes of, 145.
Milk-sickness, facts and conjectures on, 181.
Miscellaneous cases, by Dr. Hogg, 252.
Mode of preserving nitrate of silver, 468.
Monette on yellow fever, 161.
Moxa of lime, 394.
" in rheumatism, 309.
Muscular irritability, influence of nerves on, 193.
Muscles, division of for scrivener's spasm, 190.

N

Napoleon and phrenology, 399.
Natchez, yellow fever of, 161.
Nature and treatment of scrofula, 468.
Necrosis of tibia, resection, &c., 65.
Nerves, influence of on muscular irritability, 193.
New England Quarterly Journal of Medicine and Surgery, 59.
New mode of treating hydrocele, 194.
New theory of tinea, &c., 228.
Nitrate of silver, mode of preserving, 217.
Northern lakes, a summer residence for southern invalids, 401.

O

Observations on the epidemic yellow fever of Natchez, 161.
Observations on scarlatina anginosa, 327.
Operation for cataract, subconjunctival, 473.
Ourselves and our enterprise, 471.

P

Paraphymosis, treatment of, 148.
Paraplegia, ergot of rye in cases of, 395.
Permanent contraction of the fingers, 223.
Phrenology and Napoleon, 399.
Phrenological visit to penitentiary for young criminals, 233.
Physical causes of metallic tinkling, 145.
Physiological observations on double uterus, 467.
Post-mortem examination of an old soldier, 393.
Practical medicine, influence of auscultation on, 1, 80, 81.
Pregnancy, auscultation in diagnosis of, 392.
Primitive iliac artery, ligature of, 390.
Private medical instruction, 160, 239.
Professional incomes in London, 296.
Professional teachers, college of, 79.
Prolapsus and separation of the vagina, 191.

R

Rape, diagnosis of gonorrhœa in accusations of, 147.
Report on the Medical College, 235, 313.
Researches in embryology, Barry's, 257.
" into physical causes of metallic tinkling, 145.
Rupture of the diaphragm in a horse, 470.

S

Scarlatina anginosa, at St. Clairsville, Ohio, 327.
Sciatica cured by extract of belladonna, 394.
Scotland, first introduction of syphilis into, 196.
Scrivener's spasm, cured by division of muscles, 190.
Scrofula, nature and treatment of, 468.
Secondary hemorrhage after amputations, &c., 63.
Separation and prolapsus of vagina, 191.
Small-pox pustule, structure of, 219.
Southern invalids and northern lakes, 401.
Spinitis, chronic, Smith's case of, 323.
Spleen, Bourgery on anatomy of, 297.
South-west, epidemic yellow-fever of, 161.
State of American medicine before the Revolution, 149.
State of the blood in hydrophobia, 469.
Stomach, enormously enlarged, 293.
Strychnia in amaurosis, 397.
Styptic effect of creasote, 383.
Subconjunctival operation for cataract, 473.
Superior extremities, injuries of, 241.
Surgery, Liston's Elements of, 453.

T

Teachers, professional, college of, 79.
Tibia, fracture of—necrosis, resection, recovery, 65.
Times, effects of, on the profession, 189.
Tinea, new theory of—its vegetable origin, 228.
Tinkling, metallic, physical causes of, 145.
Transactions of the Alumni Society of the University of New York, 472.

Treatment of old fractures by division of tendons, 147.
Treatment of paraphymosis, 148.
Trembles and milk-sickness, 181.
Tuberculous disease, comparative frequency of, 67.
Tumors, abdominal, 306.
Typhoid and typhus fever, Bartlett on, 458.
Typhoid fever, Louis on, 386.

U

University of Virginia, 236.
Use of moxa in rheumatism, 309.
Uterus, cancerous affections of, 380.
Uterus, double, physiological observations on, 467.

V

Vagina, prolapsus and separation of, 191.
Vegetable origin of tinea, 238.
Velpeau on cysts of the eyelid, 311.
Virginia, University of 236.

W

Western Lancet, 158.
Wilson's case of compression of the brain, 321.
Womb, incipient stage of cancerous affections of, 380.
Wound of the antrum of Highmore, 186.
Wound, gun-shot, of the face, 230.

Y

Yellow fever of Natchez and the southwest, 161.

### TO DELINQUENT SUBSCRIBERS.

It has been suggested to us that the Editors of this Journal might be held responsible for the course we have taken in regard to our delinquent subscribers. Those Editors have nothing whatever to do with the business concerns of the Journal, and they have never seen any of our addresses to the subscribers until they appeared in print. They, we know now, disapprove of and protest against that course, and we must confess it was rather harsh. We shall now try the efficacy of gentlier means—we shall try what soft words will do. But we must be permitted to ask our subscribers whether they do not think we had enough to provoke us? We do not know them, we trust them on their honor, we dun them incessantly, and they neither pay nor discontinue their papers. In not ordering their papers to be stopped they admit that they are able to pay, or are base enough to take our paper without the ability to pay for it. Then, as honorable men, we must presume them able to pay, and yet many of them owe us for three years. Up to this date, after paying for editors and writers, we are losers from two to three thousand dollars by this Journal; and yet on our books are the names of 180 persons that owe us, at $15 each, $2,700. If we published this work on the cash plan, we should simply stop it; but as we commenced on the credit plan we shall go on a while longer, trusting that the consciences of our subscribers will ere long move them.

PRENTICE & WEISSINGER.

---

The Western Journal of Medicine and Surgery is published monthly by the undersigned, at the corner of Main and Fifth streets, Louisville, at $5 per annum, payable in advance. Each number contains from 80 to 84 pages making two volumes in the year of about 500 pages.

Contributions to its pages by the Physicians of the Valley of the Mississippi, addressed to the Editors, are respectfully solicited.

Letters on business, to be addressed, postage paid, to the publishers.

☞Postmasters, by the regulations of the Postoffice Department, will frank letters containing subscription money, and all remittances so franked are at the risk of the publishers.

*August*, 1842. PRENTICE & WEISSINGER.

